# 埃克替尼治疗*EGFR*敏感突变的晚期非小细胞肺癌获益患者的临床分析

**DOI:** 10.3779/j.issn.1009-3419.2016.04.04

**Published:** 2016-04-20

**Authors:** 小雯 蒋, 文娴 王, 沂平 张

**Affiliations:** 310000 杭州，浙江中医药大学附属浙江省肿瘤医院胸部肿瘤内科 Zhejiang Cancer Hospital Affiliated to Zhejiang Chinese Medicine University, Hangzhou 310000, China

**Keywords:** 肺肿瘤, *EGFR*突变, 埃克替尼, 获益者, Lung neoplasms, *EGFR* mutation, Icotinib, Benefciary

## Abstract

**背景与目的:**

靶向治疗已经成为晚期非小细胞肺癌（non-small cell lung cancer, NSCLC）治疗中不可或缺的重要手段，表皮生长因子受体（epithelial growth factor receptor, EGFR）的酪氨酸激酶抑制剂（tyrosine kinase inhibitor, TKI）可显著延长晚期携带EGFR基因突变肺癌患者生存期。埃克替尼是我国第一个拥有自主知识产权的EGFR-TKI。本研究旨在探讨埃克替尼治疗*EGFR*敏感突变的晚期NSCLC获益患者的临床特点，对获益患者[无进展生存时间（progression-free survival, PFS）≥6个月]进行回顾性资料收集并分析相关影响因素。

**方法:**

收集2011年9月1日-2015年9月30日浙江省肿瘤医院经埃克替尼片治疗的231例*EGFR*敏感突变的晚期NSCLC获益患者的生存情况。

**结果:**

经埃克替尼治疗后，一线治疗组1年获益率达67.9%，二线及以上组为53.6%，具有统计学意义（*P*=0.027）；一线治疗组2年获益率对比二线及以上组亦有统计学差异（18.7%和9.3%，*P*=0.047）。一线患者和二线及以上患者的中位PFS分别为16.7个月和12.4个月，且差异具有统计学意义（*P*=0.006）。其中有无脑转移（*P*=0.010）、埃克替尼治疗时机（*P*=0.001）、美国东部肿瘤协作组（Eastern Cooperative Oncology Group, ECOG）评分（*P*=0.001）为影响预后的主要因素。主要不良反应为皮疹51例（22.1%），腹泻27例（11.7%）。

**结论:**

埃克替尼是*EGFR*基因敏感突变的晚期NSCLC患者有效的治疗方案，其优势人群除无脑转移者及ECOG评分好的患者外，一线治疗患者疗效明显优于二线及以上者。敏感突变患者采用埃克替尼可得到较好的临床获益，并具有较好的耐受性。

## Abstract (en)

**Background and objective:**

Targeted therapy has become an indispensable therapy method in advanced non-small cell lung cancer (NSCLC) treatment. Epithelial growth factor receptor (EGFR) tyrosine kinase inhibitor (TKI) can significantly prolong the survival of patients harboring *EGFR* gene mutation. Icotinb is China's first EGFR-TKI with independent intellectual property rights. The aim of this study is to investigate the clinical characteristics about the beneficiary of advanced NSCLC patients with *EGFR* Common mutation who were treated with Icotinib. Retrospectively collect the data about beneficiary [progression-free survival (PFS)≥6 months] and analysis of the related risk factors for prognosis.

**Methods:**

From September 1, 2011 to September 30, 2015, 231 cases of advanced NSCLC beneficiary with *EGFR* common mutation were enrolled for treatment with icotinib in Zhejiang Cancer Hospital.

**Results:**

The one year benefit rate was 67.9% in the group treated with Icotinib as first line, and in the groupas second line or above was 53.6%, which is statisticallysignificant. The two years benefit rate was 18.7% and 9.3%, respectively. The median PFS of first line group and the second line or above was 16.7 and 12.4 months, respectively. The presence of brain metastasis (*P*=0.010), Prior chemotherapy (*P*=0.001), Eastern Cooperative Oncology Group (ECOG) score (*P*=0.001) were the main factors influencing the prognosis. The most common adverse were skin rashes (51 cases, 22.1%) and diarrhea (27 cases, 11.7%).

**Conclusion:**

Icotinib offers long-term clinical benefit and good tolerance for advanced NSCLC harboring *EGFR* gene mutation. Its advantage groups in addition to the patients with brain metastases and better ECOG score, the curative effect of patients with the first-line treatment is superior to second or further line.

肺癌是我国常见的恶性肿瘤之一，据《中国2011年恶性肿瘤登记年报》报告^[1]^，2011年我国肺癌发病率为48.32/10万，死亡率为39.27/10万。发病率和死亡率均居恶性肿瘤的首位。

非小细胞肺癌（non-small cell lung cancer, NSCLC）占肺癌患者的85%左右，大多数就诊时已属晚期，失去了手术治疗的机会^[2]^。据国家癌症中心全国肿瘤防治研究办公室近期在International Journal of Cancer杂志上发布我国2003年-2005年以人群为基础的癌症生存数据，肺癌5年生存率为16.1%^[3]^。两药含铂方案化疗是晚期NSCLC治疗的主要手段，其地位虽然没有发生根本改变，但疗效已达到平台期，增加化疗药物剂量只能导致毒性的增加，而不能改善生存。对晚期NSCLC的传统化疗，患者的中位生存时间约10个月左右^[4]^。同时化疗的毒副反应也限制了其广泛的临床应用。随着对恶性肿瘤发生、发展及基因、信号传导等基础研究的深入，越来越多的靶向药物应用于临床，为患者带来新的选择。靶向用药为精准治疗, 对正常组织细胞的损伤小。以表皮生长因子受体（epidermal growth factor receptor, EGFR）为靶点的药物是应用较多的肺癌分子靶向药物之一，EGFR的酪氨酸激酶抑制剂（tyrosine kinase inhibitor, TKI）显著延长晚期NSCLC患者生存期^[5]^。近年来，由于其确切的疗效、轻微的不良反应和口服给药的便利等特点，突破了传统化疗药物的瓶颈，已经成为晚期NSCLC治疗中不可或缺的重要手段。靶向药物治疗的临床研究应用成为热点之一，EGFR-TKI广泛应用于NSCLC的治疗。研究^[6, 7]^显示EGFR-TKI在二线及三线治疗中有获益。近年的临床数据^[8-11]^显示作为*EGFR*敏感突变的一线化疗在无进展生存时间（progression-free survival, PFS）方面亦有明显获益。如厄洛替尼作为治疗晚期NSCLC第一代的EGFR-TKIs药物，显示出良好的疗效及安全性^[11]^。而埃克替尼（商品名：凯美纳）是我国第一个拥有自主知识产权的EGFRTKI，也是全球第三个上市的EGFR-TKI，2011年开始在NSCLC中应用。它是一种口服制剂，在体内及体外试验中均显示出良好的活性，有良好的疗效^[12]^。Ⅲ期临床研究ICOGEN^[13]^结果显示，埃克替尼与吉非替尼疗效相近，但埃克替尼的安全性优于吉非替尼。目前已经成为国内复治晚期NSCLC治疗的标准药物之一。本文旨在回顾性分析无进展生存时间≥6个月的*EGFR*敏感突变患者的临床特征。

## 1 资料与方法

### 1.1 临床资料

2011年9月1日-2015年9月30日期间就诊于浙江省肿瘤医院的有完整随访资料并接受埃克替尼治疗的晚期*EGFR*敏感突变NSCLC患者共231例。所有病例均经组织学或细胞学确诊为晚期NSCLC且*EGFR*敏感突变，PFS≥6个月，治疗期间未采用其他抗肿瘤治疗。

### 1.2 治疗方法

所有患者均给予埃克替尼片（浙江省贝达药业有限公司）125 mg，3次/d，口服。连续服用直至疾病进展或不能耐受毒副作用为止。

### 1.3 疗效评价

患者在埃克替尼治疗开始1个月后评估疗效，疗效稳定或有效患者每2个月接受一次计算机断层扫描（computed tomography, CT）及其他影像学检查进行疗效评价。临床疗效评价参照实体瘤疗效评价标准（Response Evaluation Criteria in Solid Tumors, RECIST 1.1）进行。PFS≥6个月的患者为获益者。同时记录不良事件（adverse event, AE）和严重不良事件（serious adverse event, SAE）。

### 1.4 毒副反应

按照美国国立癌症研究所通用毒性标准4.0版评价毒副反应。

### 1.5 随访

231例患者均获得随访，患者的PFS通过门诊或电话随访获得，末次随访时间为2016年1月30日。PFS指患者自开始埃克替尼治疗到明确疾病进展的时间。

### 1.6 统计学方法

采用SPSS 19.0统计软件进行数据处理及统计分析，组间临床特征比较应用*χ*^2^检验，*Kaplane-Meier*法分析患者中位PFS，*P* < 0.05为差异有统计学意义。

## 2 结果

### 2.1 患者的一般特征

共231例患者纳入本研究。其中包括男性106例（45.9%），女性125例（54.1%）。所有患者年龄31岁-85岁，中位年龄为57岁。腺癌221例（95.7%），其他病理类型10例（4.3%）。EGFR 19外显子缺失突变139例（60.2%），EGFR 21L858R错义突变92例（39.8%）。用药前分期为Ⅲb期12例（5.2%），Ⅳ期219例（94.8%）。体能状态评分，0分-1分：188例；≥2分：43例。其中用于一线治疗134例，二线及以上治疗97例。一线与二线及以上两组患者基线资料，如性别、吸烟史、临床分期、病理类型、有无脑转移、有无肝转移、有无行颅脑放疗、EGFR基因突变位点等方面的差异均无统计学意义，组间分布较均衡（*P* > 0.05）。患者的一般特征见表 1。

**1:** 231例*EGFR*突变的患者的一般特征 Baseline characteristics of patients with *EGFR* common mutation (*n*=231)

Variables	All (*n*=231)	First line (*n*=134)	Second line and above (*n*=97)	*P*
Gender				0.686
Male	106	63 (47.0%)	43 (44.3%)	
Female	125	71 (53.0%)	54 (55.7%)	
Age (yr)				0.057
< 65	148	79 (59.0%)	69 (71.1%)	
> 65	83	55 (41.0%)	28 (28.9%)	
Smoking characteristics				0.611
Yes	72	40 (29.9%)	32 (33.0%)	
No	159	94 (70.1%)	65 (67.0%)	
Stage				0.981
Ⅲb	12	7 (5.2%)	5 (5.2%)	
Ⅳ	219	127 (94.8%)	92 (94.8%)	
Histology				0.394
Adenocarcinoma	221	130 (97.0%)	91 (93.8%)	
Non-adenocarcinoma	10	4 (3.0%)	6 (6.2%)	
Brain metastasis				0.813
Yes	59	35 (26.1%)	24 (24.7%)	
No	172	99 (73.9%)	73 (75.3%)	
Liver metastasis				0.944
Yes	17	10 (7.5%)	7 (7.2%)	
No	214	124(92.5%)	90 (92.8%)	
Mutation type				0.234
Exon 19	139	85 (63.4%)	54 (55.7%)	
21 L858R	92	49 (36.6%)	43 (44.3%)	
ECOG score				0.016
0-1	188	102 (76.1%)	86(81.4%)	
> 2	43	32 (23.9%)	11 (18.6%)	
ECOG: Eastern Cooperative Oncology Group; EGFR: epithelial growth factor receptor.				

### 2.2 一线患者及二线及以上患者的获益率比较

本研究纳入患者经埃克替尼治疗后，1年获益率一线组达67.9%，二线及以上组为53.6%，差异具有统计学意义（*P*=0.027）；2年获益率一线组对比二线及以上组分别为18.7%和9.3%，亦有统计学差异（*P*=0.047）（表 2）。

**2:** 一线及二线及以上*EGFR*突变患者临床获益率比较 Clinical benefit rate of Icotinib as fist line, second line and above in the treatment of patients harboring *EGFR* mutation

Variables DCR	First line (*n*=134)	Second line and above (*n*=97)	*P*
1 year benefit ratio	91 (67.9%)	52 (53.6%)	0.027
2 years benefit ratio	25 (18.7%)	9 (9.3%)	0.047
DCR: disease control rate.			

### 2.3 患者各临床疗效的单因素分析

本研究纳入的所有患者中位PFS为15.7个月。其中除了脑转移（*P*=0.014）、埃克替尼治疗时机（*P*=0.006）、ECOG评分（*P*=0.008）外，各组均无统计学差异（表 3）。其中凯美纳治疗前有59例脑转移患者，24例患者在凯美纳用药前或用药中接受过脑放疗，35例未接受过放疗，两组PFS无统计学差异（11.6个月*vs* 11.7个月，*P*=0.166）。一线患者的中位PFS为16.7个月，二线及以上患者的中位PFS为12.4个月，两者的差异有统计学意义（*P*=0.006）（图 1A）。虽然结果显示，19外显子缺失突变患者的中位PFS（16.3个月）略长于21外显子L858R突变患者（12.4个月），但两者在统计学上无差异（*P*=0.142）（图 1B）。

**3:** 231例*EGFR*突变的患者的PFS的单因素分析 Univariate analysis of PFS in 231 *EGFR* mutation patients

Variables	Median PFS (mon)	Range (mon)	*P*
Gender			0.226
Male	15.1	11.8-18.3	
Female	16.3	13.5-19.1	
Age (year)			0.171
< 65	15.2	12.9-17.4	
> 65	17.6	14.3-20.9	
Smoking characteristics			0.139
Yes	13.9	10.6-17.2	
No	16.3	13.8-18.8	
Histology			0.411
Adenocarcinoma	15.7	13.7-17.7	
Non-adenocarcinoma	11.0	8.6-13.3	
Brain metastasis			0.014
Yes	11.7	10.2-13.1	
No	16.3	14.5-18.1	
Liver metastasis			0.856
Yes	15.1	9.7-20.4	
No	15.7	13.9-17.5	
Mutation type			0.142
Exon 19	16.3	14.5-18.1	
21 L858R	12.4	8.9-15.8	
Treatment line			0.006
First line	16.7	14.2-19.2	
Second line and above	12.4	9.6-15.2	
ECOG score			0.008
0-1	16.3	14.7-17.9	
> 2	11.6	9.2-14.1	
PFS: progression-free survival.			

**1:**
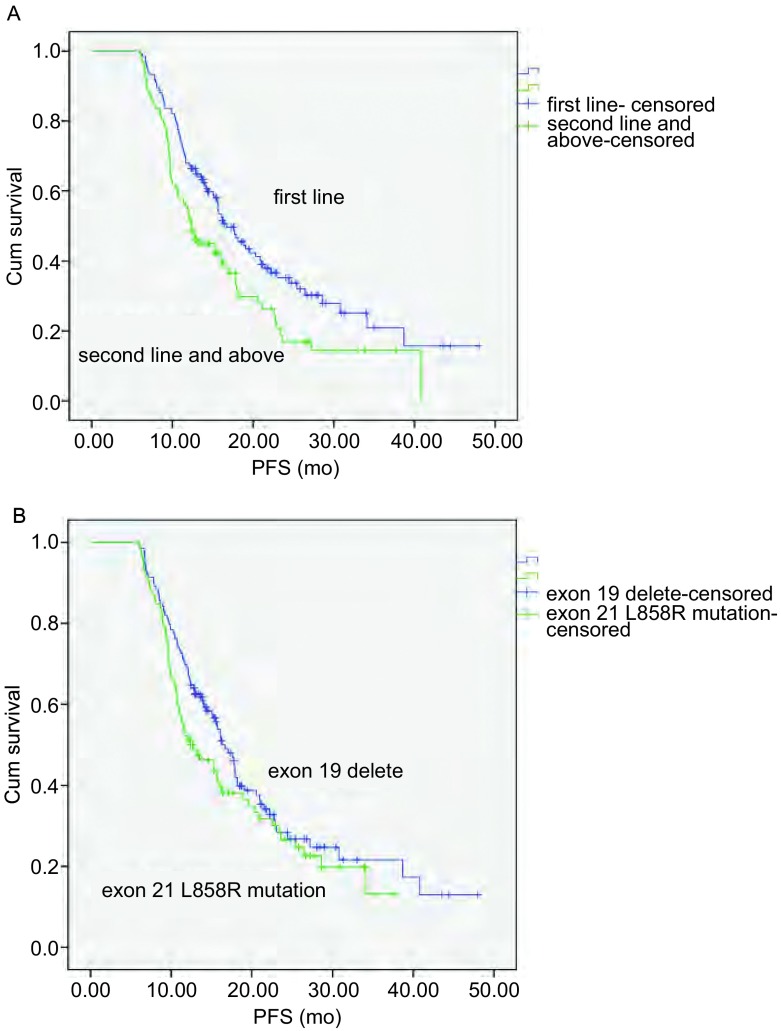
组间生存曲线比较。A：*EGFR*突变患者中一线与二线及以上患者的PFS比较（16.7个月*vs* 12.4个月，*P* =0.006）；B：EGFR外显子19缺失患者与外显子21 L858R突变患者的PFS比较（16.3个月*vs* 12.4个月，*P* =0.142）。 Comparison of survival curves between groups. A: PFS of *EGFR* mutation patients with first line *vs* second line and above (16.7 months *vs* 12.4 months, *P* =0.006); B: PFS in EGFR exon 19 delete and exon 21 L858R mutation patients (16.3 months *vs* 12.4 months, *P* =0.142).

### 2.4 埃克替尼治疗后的PFS的*Cox*多因素分析

将单因素分析中有意义的因素：脑转移、埃克替尼治疗时机、ECOG评分进入多因素*Cox*风险比例模型分析显示：有无脑转移（*P*=0.010）、埃克替尼治疗时机（*P*=0.001）、ECOG评分（*P*=0.001）为影响预后的主要因素（表 4）。

**4:** 231例*EGFR*突变的患者的PFS的*Cox*多因素分析 *Cox* regression analysis of PFS in 231 *EGFR* mutation patients

Variables	OR	95%CI	*P*
Brain metastasis *vs* no Brain metastasis	0.636	0.451-0.896	0.010
First line *vs* Second line and above	1.692	1.227-2.334	0.001
ECOG score 0-1 *vs* ECOG score≥2	1.926	1.290-2.876	0.001

### 2.5 安全性评估

经长期埃克替尼患者的安全性较好，治疗过程中主要毒副反应为皮疹和腹泻。纳入的231例患者中，共51例（22.1%）患者发生皮疹反应，其中Ⅰ度44例，Ⅱ度7例。27例患者发生腹泻，发生率为11.7%，其中Ⅰ度23例，Ⅱ度4例。Ⅰ度-Ⅱ度肝功能异常发生率19.5%。其他不良反应包括恶心、食欲不振等，多为Ⅰ度-Ⅱ度，未见Ⅲ度-Ⅳ度不良反应。所有患者无一例因毒副反应而停止盐酸埃克替尼治疗（表 5）。

**5:** 231例*EGFR*突变的患者的不良反应 Adverse events of patients with *EGFR* common mutation (*n*=231)

Adverse events	*n*	Grade Ⅰ	Grade Ⅱ	Grade Ⅲ-Ⅳ
Rash	51	44 (19.0 %)	7 (3.0%)	0
Diarrhea	27	23 (10.0%)	4 (1.7%)	0
Raised aminotransferase	45	34 (15.0%)	11 (4.8%)	0
Nausea	12	12 (5.2%)	0	0
Loss of appetite	14	14 (6.0%)	0	0

## 3 讨论

肺癌的高发病率、高病死率均居于癌症首位，近年来，肺癌化疗的疗效并未取得突破性进展，而EGFRTKI使晚期NSCLC患者生存期显著延长^[5]^。近10余年来，EGFR-TKI的研究已经取得了很多里程碑意义的结果。多项大型的国际多中心临床研究^[10, 11, 14]^均已证实：对于*EGFR*敏感突变的晚期NSCLC患者，EGFR-TKI治疗的PFS及客观缓解率（objective response rate, ORR）均优于传统细胞毒药物的化疗。而且EGFR-TKI不仅显著延长晚期NSCLC患者生存期，研究^[15]^还发现EGFR-TKI治疗进展后继续使用并联合化疗或放疗较单纯化疗或放疗可显著延长患者的总生存期（overall survival, OS）。EGFR-TKI主要包括吉非替尼、厄洛替尼和埃克替尼。埃克替尼是我国完全拥有知识产权的小分子靶向药，也是继厄洛替尼和吉非替尼后国际上第3个EGFR-TKI药物，与其他两种药物相比，自2011年上市以来临床应用时间尚短。

第一个头对头比较埃克替尼与吉非替尼疗效的Ⅲ期临床研究ICOGEN^[13]^显示，在复治晚期NSCLC中，埃克替尼疗效不劣于吉非替尼，患者的PFS较吉非替尼延长35.3%，其中*EGFR*突变型的患者的PFS较吉非替尼延长24.5%，而OS、疾病进展时间（time to progression, TTP）、ORR、疾病控制率（disease control rate, DCR）方面与吉非替尼相当，且埃克替尼毒副反应低于吉非替尼。但该研究共入组399例患者，其中仅29例具有*EGFR*突变的患者服用埃克替尼。Hu等^[16]^对埃克替尼治疗晚期NSCLC安全性的多中心四期研究中，纳入的5, 549例患者中*EGFR*突变患者665例，ORR和DCR分别高达49.2%和92.3%。两个研究结果均说明，埃克替尼治疗NSCLC人群可获得良好的疗效及安全性。

IPASS研究^[8]^奠定了EGFR-TKI在晚期NSCLC患者中一线治疗的地位。在*EGFR*突变患者当中，吉非替尼组ORR对比化疗组为71.2%和47.3%。随后几项大型研究^[9-11, 14]^均通过EGFR-TKI一线治疗对比化疗，证实了其一线治疗在*EGFR*突变患者中的疗效。基于上述研究结果，2010年美国和中国的肿瘤临床指南均明确指出，推荐*EGFR*基因突变的晚期NSCLC患者一线使用EGFR-TKI。本研究对纳入的231例*EGFR*突变患者中一线及二线及以上获益患者疗效进行分析，结果显示，埃克替尼一线治疗与二线及以上的患者均能从中获益，但一线患者的PFS明显高于二线及二线以上（16.7个月*vs* 12.4个月，*P*=0.006）。埃克替尼一线治疗组1年获益率和二线及以上组分别为67.9%和53.6%；2年获益率分别为18.7%和9.3%，均有差异（*P* < 0.05）。本研究还观察到，ECOG评分是PFS的影响因素，ECOG评分为0分-1分的患者PFS长于≥2分（16.3个月*vs* 11.6个月，*P*=0.008），对于ECOG评分差的患者，使用埃克替尼治疗即使初始有效，其PFS仍不如ECOG评分良好者。值得关注的是，本文对患者有无脑转移做了统计，结果发现无脑转移患者的PFS较有脑转移患者更长（16.3个月*vs* 11.7个月，*P*=0.014）且有统计学差异。59例脑转移患者有24接受脑放疗，35例未接受放疗，两组PFS无统计学差异（11.6个月*vs* 11.7个月，*P*=0.166），因此，对于无症状的脑转移患者，可以不加用放疗或不首选考虑同步脑放疗。黄江等^[17]^在NSCLC患者生存3年以上的因素分析中提示ECOG评分、临床分期对3年生存率均有显著影响。Park等^[18]^报道采用厄洛替尼治疗28例*EGFR*突变型的NSCLC脑转移患者，中位PFS为6.6个月（95%CI: 3.8-9.3）。Porta等^[19]^报道厄洛替尼治疗*EFGR*突变型NSCLC脑转移患者的中位PFS为11.7个月（95%CI: 7.9-15.5），张贝贝等^[20]^报道埃克替尼治疗NSCLC脑转移的回顾性研究中，*EGFR*突变型为10.1月，野生型为4.5个月，本研究结果与以上报道结果相近。

在安全性方面，本研究显示，埃克替尼的不良反应以皮疹和腹泻为主，发生率和既往ICOGEN及近期报道^[21, 22]^相似，但低于吉非替尼和厄洛替尼的不良反应发生率^[6]^。说明埃克替尼的耐受性较好，为体力状态差、年龄大、不能耐受化疗的晚期NSCLC患者提供了治疗机会。

关于长期获益的预后相关风险因素，已有临床研究表明：EGFR-TKI对于*EGFR*突变的优势人群：女性、腺癌和非吸烟患者的疗效较好^[8, 23, 24]^。本研究显示中老年男性、非腺癌及吸烟患者在埃克替尼的治疗中均有获益，且一线患者、无脑转移者及ECOG评分好的患者疗效较好。*Cox*多因素风险评估亦显示：有无脑转移（*P*=0.010）、埃克替尼治疗时机（*P*=0.001）、ECOG评分（*P*=0.001）为影响预后的主要因素。外显子19缺失突变和外显子21 L858R突变是目前最主要的两种突变类型。LUX-Lung 3研究的亚组分析^[25]^提示二代EGFR-TKI治疗*EGFR*敏感突变患者，中位PFS及中位OS在EGFR 19外显子缺失突变患者中较化疗组显著延长，而在EGFR 21错义突变患者中虽然有相对延长但无统计学差异。谢亚琳等^[26]^在一代EGFR-TKI吉非替尼与厄洛替尼治疗*EGFR*敏感突变患者中显示，19号外显子缺失患者的PFS比21号外显子L858R错义突变型患者的相对长，但两组之间PFS无统计学差异（*P*=0.072）。TRIBUTE^[27]^研究亚组分析得到的结果也提示*EGFR*突变状态与疗效有关。与上述研究结果相似，本组研究提示19外显子缺失突变患者的PFS长于21 L858R错义突变患者（16.3个月*vs* 12.4个月），但是两者在统计学上仍然无差异（*P*=0.142），可能跟肿瘤存在异质性有关，这有待进一步的相关研究证实。目前尚缺乏对靶向药物治疗EGFR不同敏感突变之间的疗效作头对头的临床研究。

由于本研究为回顾性研究，可能存在一定的局限性。随着现代基因检测水平不断发展及靶向用药前的基因检测日渐受到重视，仍需要对*EGFR*基因不同敏感突变状态之间的大样本临床研究。

综上所述，埃克替尼是治疗*EGFR*敏感突变的晚期NSCLCL患者的有效方案，其优势人群除无脑转移者及ECOG评分好的患者外，一线治疗患者疗效明显优于二线及以上者。无论19外显子缺失突变还是21外显子L858R错义突变均可以从EGFR-TKI治疗中获益。
